# Exploration of the vein to vein blood transfusion system in three Kenyan counties: a qualitative study

**DOI:** 10.1016/j.langlo.2026.103968

**Published:** 2026-06-13

**Authors:** Linda S Barnes, Jennifer Makin, Jose R Paiz, Jana Macleod, Cindy Mical Makanga, Tecla Kivuli Chelagat, Alejandro Munoz-Valencia, Ejemai Eboreime, Robert Kamu Kaburu, Abdirahman Musa, Carolyne Njoki, Cavin Otieno Opiyo, Kristina E Rudd, Mark Yazer, Bopaya Bidanda, Juan Carlos Puyana, Pratap Kumar

**Affiliations:** Linda S Barnes Consulting, Seattle, WA, USA; University of Pittsburgh, Pittsburgh, PA, USA; Strathmore University, Nairobi, Kenya; University of Pittsburgh, Pittsburgh, PA, USA; University of Pittsburgh, Pittsburgh, PA, USA; Strathmore University, Nairobi, Kenya; Kenyatta University, Nairobi, Kenya; Strathmore University, Nairobi, Kenya; Strathmore University, Nairobi, Kenya; University of Pittsburgh, Pittsburgh, PA, USA; Dalhousie University, Halifax, NS, Canada; Strathmore University, Nairobi, Kenya; Strathmore University, Nairobi, Kenya; Aga Khan University, Nairobi, Kenya; Strathmore University, Nairobi, Kenya; University of Pittsburgh, Pittsburgh, PA, USA; University of Pittsburgh, Pittsburgh, PA, USA; University of Pittsburgh, Pittsburgh, PA, USA; University of Pittsburgh, Pittsburgh, PA, USA; Royal College of Surgeons, Dublin, Ireland; Strathmore University, Nairobi, Kenya; University College London, London, UK

## Abstract

**Background:**

Severe shortages of blood for transfusion are a persistent global health crisis, with the burden disproportionately concentrated in low-income countries. Diverse stakeholder perspectives on blood availability might inform improvements. We aimed to use a comprehensive approach to identify the challenges, facilitators, and innovations within the blood transfusion continuum (BTC) in three different Kenyan settings.

**Methods:**

In this qualitative study, we interrogated the BTC in Siaya, Nakuru, and Turkana counties as part of the Pathways for Innovation in Blood Transfusion Systems in Kenya (PITS Kenya) mixed methods research study. We used purposive sampling, and through interviews and focus group discussions, we solicited perspectives about the challenges, barriers, and facilitating features of the BTC, including any innovations, from health-care workers, hospital administration, government officials, transfused patients, and blood donors aged 18 years and older, who were familiar with the BTC, from Aug 5, 2021, to May 9, 2022. We thematically analysed interview and discussion transcriptions using an iterative approach to achieve saturation of the thematic content. The findings were validated via member checking and stakeholder meetings in Nairobi that included research participants, non-governmental organisations, and Kenyan health authorities.

**Findings:**

197 participants (84 [43%] female and 113 [57%] male) participated in 74 interviews (76 participants) and 18 focus group discussions (121 participants). Seven themes emerged: (1) massive shortages of blood, (2) mismatches between where blood is stored and where patients need it, (3) reliance on family replacement donors, (4) delays in patient care, (5) stresses to the health-care system and workforce hardship, (6) variable knowledge and attitudes towards blood donation and transfusion, and (7) limited resources. Facilitation of blood transfusion involved local champions through self-organised and community engagement. Innovations advocated for improved resource allocation, education, and collaborative efforts.

**Interpretation:**

This study identified BTC challenges, facilitators, and innovations. The participants described the BTC as fragmented in these settings. The most poignant findings revealed how the lack of blood availability caused profound stress on the health system, contributing to delays, inefficiencies, and worry for patient wellbeing. Innovations encouraged collective stewardship and governance of the BTC, bridging communities and health-care facilities.

**Funding:**

The US National Institutes of Health National Heart, Lung, and Blood Institute.

## Introduction

Blood transfusions enable essential medical care, reducing morbidity and mortality for patients affected by chronic anaemia to acute haemorrhage. 61% of countries do not have adequate blood supply to deliver this care, with an estimated shortage of 102 million red blood cell units annually.^1,2^ Low-income and middle-income countries experience the brunt of the deficit.^2^ Nearly every country in sub-Saharan Africa faces chronically severe blood shortages.^3–5^ Blood availability depends upon a reliable donor population and the ability to process, test, store, and deliver blood, along with the use of evidence-based transfusion practices for patient care. This so-called vein-to-vein series of steps, from blood donation to transfusion, comprise the blood transfusion continuum (BTC).^1^

Previous research describes many factors contributing to insufficient blood availability in Kenya, including high rates of chronic anaemia and transfusion transmitted infections, community fears and mistrust, low levels of voluntary blood donation, and reliance on school-aged children for blood drives.^6^ Other challenges include complex logistics associated with storage, transportation, testing, and processing of blood.^7^ Hospital staffing and supply deficits also impair timely blood transfusion.^8,9^ Few studies detail the perspectives of health-care professionals who encounter daily difficulties fulfilling the clinical demand for blood transfusion at the hospital level, or the broader view of community stakeholders and health system authorities engaged in the BTC.

We sought to explore diverse stakeholder viewpoints using qualitative research methods in three Kenyan counties: Siaya, Nakuru, and Turkana. The objectives were to gather information on the challenges and opportunities in providing a sufficient and reliable blood supply from health-care, government, and community members experienced in the BTC, considering benefits and harms. We hypothesised that health-care workers, hospital administration, government officials, transfused patients, and blood donors were best situated to (1) describe critical challenges in blood donation, processing, and delivery; (2) identify facilitators contributing to possible improvements; and (3) inform novel innovations to close gaps in blood transfusion availability. Given the complexity of the BTC, we anticipated that a holistic view would expose opportunities for increased performance of the system overall.

## Methods

### Study design

We conducted a qualitative study using an exploratory design of the Kenyan blood transfusion system as part of the ongoing Pathways for Innovation in Blood Transfusion Systems in Kenya (PITS Kenya) mixed methods research study that started on July 1, 2020.^10^ We obtained Ethical Review Board approval from Strathmore University (Nairobi, Kenya; SU-IERC0992/21), the National Commission for Science, Technology and Innovation (Nairobi, Kenya; NACOSTI/P/21/9976), the University of Minnesota (Minneapolis, MN, USA), and the University of Pittsburgh (Pittsburgh, PA, USA; STUDY21020040). Our previously published protocol paper describes the design, setting, and metatheoretical framework.^10^

### Participants

We used a purposive sampling method to guide participant selection across the range of the BTC. We applied a sampling matrix that targeted representatives from each stakeholder category to ensure balanced representation from multiple levels of the health system, officials, and community members (appendix 1 p 1). We sought at least 120 adults with experience in the BTC within Nakuru, Turkana, and Siaya counties to include in the study. We purposefully selected health-care facilities within the targeted counties to include both smaller peripheral hospitals and larger referral centres. Study personnel approached prospective participants in three separate ways, with consideration given to potential for adverse impact or harm. (1) We contacted clinicians and administrators within the selected facilities considering their professional roles within the BTC through department rosters and directly invited their participation. We selected various clinical specialties and officials to capture a full spectrum of the BTC across the health-care system. (2) We invited transfused patients through ward census reviews and approached adults (aged ≥18 years) capable of consenting after their acute clinical needs resolved. (3) We asked for blood donors through managers at satellite blood donation centres and referrals to community blood donor groups. This multilevel and role-based sampling approach ensured representation of BTC perspectives across the health system, community, and government.

### Procedures

Interview tools consisted of a semi-structured guide with questions informed by the Consolidated Framework for Implementation Research (CFIR).^11^ The topics explored, in the form of open-ended questions, are summarised in appendix 1 (pp 2–3). The questions and prompts focused on the participant’s perspective and experience within the BTC to acquire their perceptions of challenges, barriers, and enabling features, including any innovations that each participant might offer. For an innovation, participants were asked about acceptability, feasibility, and appropriateness within their setting. The tools were developed by blood transfusion experts and clinicians with practical experience in Kenya (JMak, JMac, MY, PK, and JCP). We pilot tested the questions with nine research team members, health-care workers, and community representatives in Kenya, who were uninvolved in the qualitative part of the research. We refined the tools before starting the formal study by clarifying the questions and prompts. As well as individual interviews, we conducted focus group discussions, which involved community members (blood donors) or clinicians (medical providers or nurses).

Three teams of trained research staff, one per county, performed data collection. Each team consisted of a team leader and two research assistants. Participants gave individual written consent in either English, Swahili, Luo, or Turkana. De-identified recordings, restricted to the research team, were uploaded to Otter.ai and transcripts were downloaded to secure institutional storage. Files were removed from Otter.ai after transcription in compliance with the study’s data management procedures.^10^ For interviews conducted in Swahili, Luo, or Turkana, research team members (TKC, CMM, RKK, and AM) fluent in these languages transcribed the audio and then translated them into English. All transcriptions were verified against the audio recording and minimally refined by a second member of the research team to improve linguistic clarity, remove vocal disfluencies or hesitations, and incorporate verbal emphases that were not evident in the transcripts alone.

### Data analysis

The qualitative analysis followed a structured and iterative approach focused on thematic domains without predefined outcomes (appendix 1 p 4). To provide consistent interpretation of participant voices, a codebook captured key constructs from the CFIR conceptual framework and definitions. The research team (LSB, TKC, AM, CMM, JMac, JMak, AM-V, and PK) augmented this initial codebook by reviewing the literature before data analysis, then improving the definitions through a workshop that incorporated a debrief of field notes to ensure relevance and applicability. We performed a preliminary analysis on a selection of 15 transcripts, five from each county, to refine the codebook and definitions. The researchers (LSB, TKC, CMM, JMac, JMak, AM-V, and PK) met three times to resolve differences in application of codes allowing for further refinement of the codebook, definitions, and incorporation of emerging concepts informed by the transcribed data. Subsequent rounds of analysis included the entire body of 92 transcripts.

We used computer-assisted qualitative data analysis software MAXQDA for data analysis. Six researchers (LSB, TKC, CMM, AM-V, JMac, and JMak) trained in qualitative research performed the analysis using a systematic, thematic approach to identify and interpret key content and patterns related to the BTC. The researchers reviewed interview transcripts repeatedly to gain familiarity with the data. To assess intercoder reliability, we conducted paired analysis on a series of transcripts and resolved the discrepancies through interactive discussions to achieve high concordance. We applied an inductive approach to the initial conceptual framework guided by the coding process and a deductive approach to iteratively incorporate new insights, allowing for both a priori and emergent themes to surface. Memos summarising contemporaneous researcher impressions of the data informed emergent codes, patterns, and themes. The process of identifying emerging themes, with data coded into broad categories, led to the refinement of specific subthemes.

Summary themes were developed for constructs and subconstructs that were related to facilitators, barriers, and innovations, then were exported to Microsoft Excel. The themes were organised into tables to provide a comprehensive overview of the findings and implications. The final stage of analysis involved synthesising these themes into actionable recommendations aimed at improving the BTC. Throughout the analysis, attention was given to the context of the collected data, and themes were cross-checked for consistency across the types of data (recordings, memos, and transcripts). The research team discussed the themes from the preliminary findings with participants during structured site visits and conducted an external validation of the emerging themes at a stakeholder meeting held in Nairobi comprising research participants, non-governmental organisation partners, and health authorities. These activities occurred before comprehensive analysis and peer debriefing with other knowledgeable experts in the BTC. The comprehensive analysis involved regular research team meetings to achieve thematic saturation of the concepts conveyed by participants, address potential biases, and ensure the accuracy of interpretation.

### Role of the funding source

The funder of the study had no role in study design, data collection, data analysis, data interpretation, or writing of the report.

## Results

Between Aug 5, 2021, and May 9, 2022, 197 participants (84 [43%] female and 113 [57%] male) took part in 74 interviews (76 participants) and 18 focus group discussions (121 participants; table). Individual interviews ranged from 10 min to 141 min. Focus group discussions, each comprising five to eight participants by discipline, included medical officers, clinical officers, inpatient and outpatient nurses, and blood donors, and ranged in duration from 35 min to 123 min. The recordings captured 4669 min of content.

Seven overarching themes arose from the data, detailing critical challenges and barriers contributing to the lack of blood availability in the three counties (panel 1). (1) Massive deficit in blood availability.

“Today there is blood and tomorrow we don’t have any.” *Focus group discussions, clinicians, 131110*

(2) Mismatch between where blood is stored and where it is needed, reflecting supply chain challenges. (3) An impractical reliance on family replacement donors. (4) Blood shortages impact patient care and lead to poor health outcomes; donors, patients, and health-care workers frequently discussed prolonged hospital stays, not transfusing the desired amount of blood required for the degree of anaemia and haemostasis, or patient safety, or both. (5) Concern for patient outcomes causes stress to the health-care system. (6) Knowledge, attitudes, perceptions, and beliefs about transfusion and donation differ by site and influence the willingness of donors to donate blood and providers to prioritise patients for transfusion. (7) Lack of equipment and resources limits the ability to collect, process, store, transport, and transfuse blood.

The adverse consequences of these challenges ranged from creating a strain on the health system, health-care workers, patients, and their families to a low public willingness to donate and a lack of resources. These themes illustrate the complexity of governance over the BTC and the fragmented yet interconnected nature of blood supply and transfusion demand.

Multiple factors contributed positively to the functioning of the BTC, both in the health-care facility and community setting (panel 2). A recurring facilitator across all sites was the motivation and perseverance of the health-care workforce. Health-care workers and administrators described having to push hard to hunt for blood, even seeking blood in the community for emergencies.

“I went to a nearby school. I went to the principal, and I requested…give me only five students who can donate blood…it was screened [tested] then I took it by myself to ICU [intensive care unit].” *Interview, administration, 1301094*

In many instances, health-care workers personally visited the laboratory to advocate for their patients, called other facilities to locate blood, or sourced petrol and vehicles to transport necessary blood products. On a routine basis, health-care workers described educating patients and their families regarding the safety and need for blood donations. Teamwork between departments and across facilities contributed to successful transfusions. Individuals created informal supply chains between public and private facilities and blood banks. Family replacement donors and individuals with relationships to their patients described a compelling motivation to donate blood. Close physical proximity to the facilities capable of collecting and testing blood appeared vital, and facilities without these resources struggled with transportation delays. Reliance on schools as blood drive locations contributed high yields of willing donors when in session. Notably, community governance interactions allowed for cocreation of campaigns from stakeholders through multisector collaboration, and social accountability mechanisms that engaged the communities with the need for blood transfusion in the local health-care settings.

Participants identified innovations and adaptations across the BTC (panel 2). The most common innovation was adapting to scarcity.

“Blood is never enough, but we’ve learnt over years to work with what you can get. And most of the times…you end up doing without it.” *Focus group discussions, clinicians, 131060*

Common suggestions included community education and empowerment, such as advocating for donations from community leaders, educating the public to reduce fear of donating, and promoting healthy behaviours and lifestyles. A specific example of successful community mobilisation came from a sports club in Turkana, where members organised and participated in multiple blood drives. Many participants emphasised the need to implement quality standards and develop a governance system for estimating the region’s blood supply and demand. Fluctuations in peak demand, during seasonal malaria, and low blood donation periods, such as school holidays, were highlighted. A proposed strategy was to establish databases to track repeat blood donors to enable their re-engagement and donation when needed, particularly through digital platforms such as WhatsApp and Damu Sasa. Several participants also advocated increasing the number of regional blood drives and increasing convenient public access points to donate blood conveniently. Additionally, many suggested replicating the example seen in Nakuru by establishing local blood donation centres able to perform blood collection and on-site processing and testing of blood products within the hospital facility.

“Our facility is a bit different we have a blood bank, a small one, so if we need [blood], especially [for] the patients in the ward that can wait, we ask the relatives to come and donate, they come, they donate.” *Focus group discussions, clinicians, 131086*

Participants hypothesised that bringing blood donors and the resultant blood products closer to recipients reduced transportation costs and the risk of delays. Overall, these innovations aim to address the fragmented nature of the BTC and better align the blood supply with the demand for transfusions. These examples showed that integration of community stewardship for the blood supply, including self-organised campaigns, multisector collaboration, and social accountability to align stakeholders.

The research team sorted and refined the emerging themes in panels 1 and 2 to develop a conceptual framework to articulate the recommendations for improving the BTC (figure). Within this figure, we categorised the overarching themes comprising challenges, the contextual innovations, and facilitators of blood transfusion availability. To improve the BTC, participants recommended strengthening the linkages between communities and health-care facilities through more coordinated and collaborative actions to balance available blood supplies. Additionally, resources to overcome limitations in equipment, transportation, and workforce constraints are important. These innovations aim to enhance the overall efficiency of the BTC by closing existing gaps and ensuring a more integrated, responsive system.

## Discussion

Through the voices of transfused patients, blood donors, health-care professionals, and governing officials in three diverse Kenyan locations, this qualitative study shows that inadequate blood availability is not only a profound and pervasive problem of insufficient blood donation, but a fragmented BTC in which weak linkages between community mobilisation and local health-care facilities yield delays, inefficiencies, and distress across the entire system. Our methods led to a deep contextual understanding, informing a conceptual framework to collectively overcome challenges, thus thwarting the expectation of scarcity or desperate helplessness.^12,13^ Participants described both established and novel solutions, their perspectives on acceptability, and the barriers and facilitators to implementation. Overall, the findings provide a comprehensive view of the current state of the BTC in Kenya and suggest actionable pathways for future progress, meeting the three primary objectives of identifying key challenges, facilitators, and innovations.

Importantly, the participants in this study perceived the BTC as discontinuous in these settings. The community connects to the blood bank through desperate individuals pressing for semi-structured, self-organised donor mobilisation activities, such as family replacement donors or infrequent blood drives, while blood banks maintain unsteady linkages to hospital laboratories where clinical demands for transfusion are acutely arbitrated, patient by patient, considering exhausted blood inventories. This fragmentation between the community and health-care facilities creates gaps in the supply and demand for blood, making effective mediation of blood availability difficult. The challenges of the BTC in Kenya were consistent with known barriers of limited infrastructure and resources.^14^ Participants acknowledged the difficulty in securing adequate and sustainable support to strengthen the BTC. Yet no single discipline or stakeholder category was able to fully articulate the complexity of the problem. The prevailing impression revealed a fractured system characterised by mismatches between supply and demand described as disruptions or delays between where blood is available and where it is most needed. There was a general sense of mistrust and exhaustion among participants, stemming from their own observed challenges, misinformation, and broader knowledge gaps, which contributed to this concept of fragmentation. The overarching theme across all interviews was that blood availability consistently failed to meet the demand for transfusions, aligning with the inadequate blood supply documented across sub-Saharan Africa.^15–17^

Participants described facilitation of blood availability as being enabled by individual stakeholders, advocates, or local champions who acted as pillars supporting the BTC. Other publications also found local champions to be vital catalysts.^18,19^ However, we found dependence on singular advocates to be both a facilitator and a barrier when each stakeholder exists in isolation. A health-care worker who leverages political influence or creates informal supply chains is a facilitator; however, their reliance on personal effort and the risk of health-care worker burnout underscores the limitations of depending on individual will. Our data suggest there are inordinate pressures and indirect costs put on families of family replacement donors. For example, a family replacement donor who answers the call to donate blood and mobilises their network is an important facilitator to motivate blood donation directed to a patient. Like findings from other settings, reliance on this method of blood supply in this region has proven unsustainable because the need for blood transfusion might be urgent and precede blood donation (eg, trauma or haemorrhage).^20^ The families and patients expressed resentment and the hospital staff and patients were desperate to receive timely blood for emergent transfusion. Participants advanced a systems-thinking approach that fostered coordination, collaboration, and partnership across the BTC.^21^ We suggest moving away from sole reliance on individual champions or family replacement donors towards developing sustainable system level supports such as dedicated transfusion coordinator roles, blood demand forecasting tools, and interhospital blood sharing platforms.^22,23^ Policies ideally reinforce coordinated governance across national, county, facility, and community levels, with clear roles and accountability mechanisms. Embedding structured, community-led approaches within policy frameworks might enhance sustained blood donation and better align local participation with system needs.

Emerging innovations identified modern technologies to increase awareness, including digital and social media platforms. The innovations sought transparency and shared ownership, connecting the community and hospital with the mutual governance of the BTC, to overcome barriers and improve blood availability. Participants described the need for interoperable digital systems enabling real-time data sharing on donor availability, blood stocks, and demand forecasting, as well as investments in systems level change, including resourcing local blood testing and processing capabilities. The emphasis on expanding community-based blood donation and blood drives generated the most enthusiasm and support from the stakeholders. Community engagement has been a core pillar of blood transfusion improvement endorsed by WHO Regional Office for Africa strategies since 2001 that call for community-based mobilisation and voluntary blood donation.^24^ Community walking blood banks such as those implemented in Turkana County might help to close emergency gaps in blood availability.^25^ However, the most universal, sustainable, and powerful solutions, according to the participants, leveraged teamwork, networking, and civic engagement, fostering stewardship of the BTC to fulfil a sufficient blood supply for patients requiring blood transfusion.^26,27^ For blood donation to take on real, practical significance, it must become an integral part of the community.^13,28,29^ Participants articulated the importance of connecting and uniting each component of the BTC over optimising any single aspect or working heroically in isolation.

Limitations of this study include that, despite the large number of participants and diversity of their perspectives, the purposeful sampling might not represent all perspectives on the BTC. Further, participants might have provided what they thought was socially desirable information, contributing to bias. Participants might also have been biased by their most recent or worst experience with blood transfusion or donation, and this recall bias could have affected their answers and general opinion of the BTC. Measures taken to reduce researcher bias included paired coding comparisons, memoing (documenting contemporaneous impressions), and refining definitions through a group process that involved peer debriefing, member checking, and stakeholder input. Future research might facilitate the transferability of these findings beyond the settings included in the study. This study does not account for changes over time in the study settings.

Despite their challenges within the BTC, stakeholders worked tirelessly. The resilience and passion of these individuals and their cooperative efforts offer optimism for a stronger, more robust BTC in the future. Although many of the innovations identified by participants would undoubtedly benefit the system, no single technology or solution was viewed as a panacea.^6,25^ The participants’ vision of the BTC underscores that achieving an adequate blood supply demands more than technical solutions; it depends on innovation that bridges communities and health-care delivery. Collective stewardship and shared governance of the BTC are essential to reliably realising a safe, sufficient, and sustainable blood supply from vein to vein.

## Supplementary Material

supplemental appendix 2

supplemental appendix 1

## Figures and Tables

**Figure:**
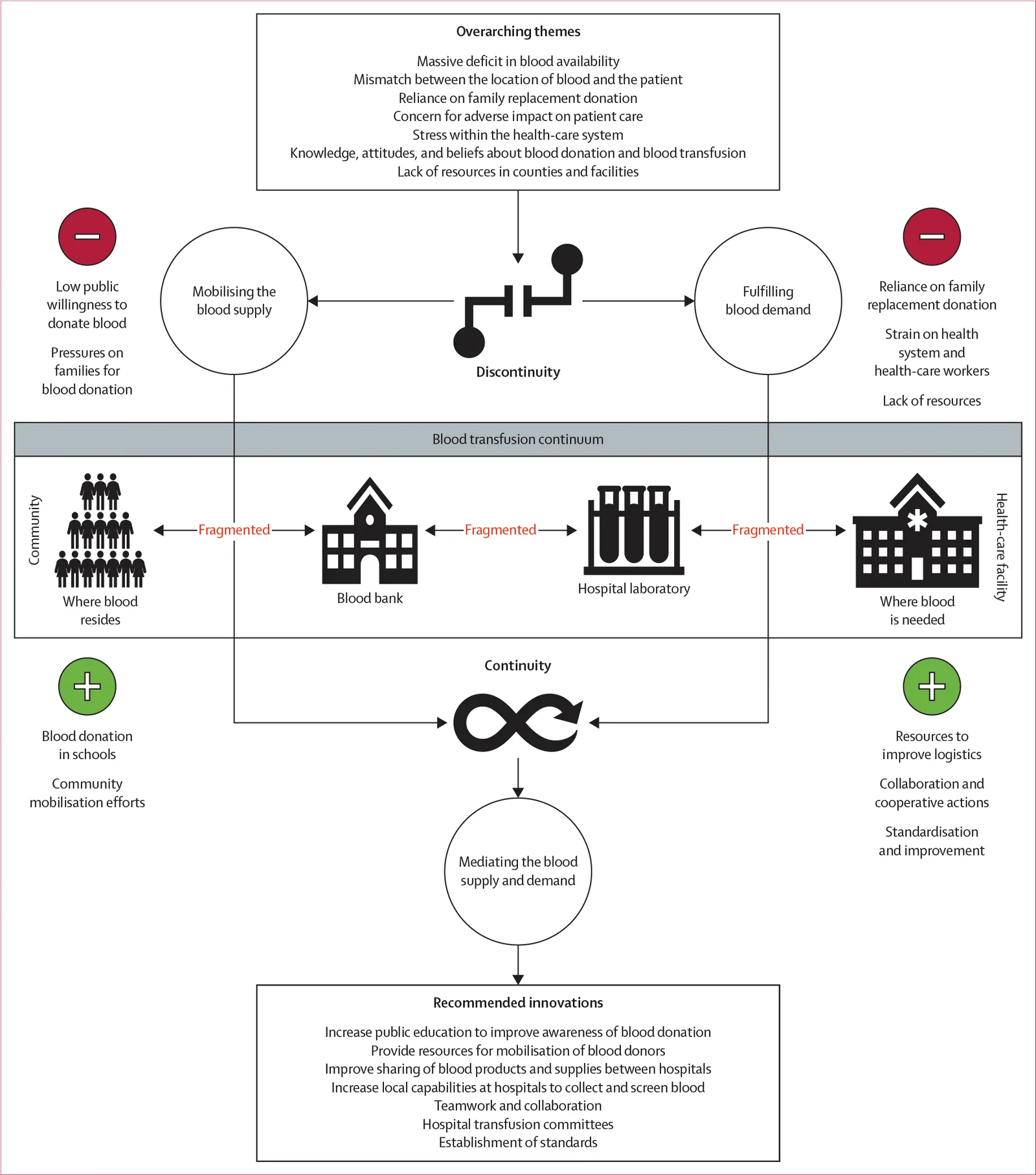
Blood transfusion conceptual framework The participants described the blood transfusion continuum as discontinuous and fragmented where weak linkages between community mobilisation, blood banking, transport, hospital laboratories, and bedside clinical care generate delays, inefficiencies, and distress across the system. Impediments (red icon) and facilitation (green icon) in mobilising the blood supply and fulfilling clinical demand for blood pointed towards continuity across the blood transfusion continuum. Increased connectivity between the community and health-care facilities aids effective mediation of the blood supply and demand for blood transfusion.

**Table:** Participant characteristics

	All participants (n=197)	Key informant interviews (n=76)	Focus group discussions (n=121)

Sex			
Female	84 (43%)	25 (33%)	59 (49%)
Male	113 (57%)	51 (67%)	62 (51%)
Age, years	34 (28–41)	40 (34–47)	30 (28–35)
Employment status			
Student	10 (5%)	2 (3%)	8 (7%)
Unemployed or retired	10 (5%)	3 (4%)	7 (6%)
Homemaker	4 (2%)	4 (5%)	0
Self-employed or part-time employment	25 (13%)	17 (22%)	8 (7%)
Full-time employment	148 (75%)	50 (66%)	98 (81%)
County of residence			
Turkana	65 (33%)	23 (30%)	42 (35%)
Nakuru	64 (32%)	25 (33%)	39 (32%)
Siaya	65 (33%)	25 (33%)	40 (33%)
Nairobi	3 (2%)	3 (4%)	NA†
Stakeholder role			
Patient or family member	27 (14%)	27 (36%)	0
Blood donor	38 (19%)	NA*	38 (31%)
Health system	8 (4%)	8 (11%)	NA†
Hospital	20 (10%)	20 (26%)	NA†
Blood bank	4 (2%)	4 (5%)	NA†
Medical provider or clinician	100 (51%)	17 (22%)	83 (69%)

Data are n (%) or median (IQR). Percentages might not add up to 100% due to rounding. NA=not applicable.

* All stakeholders in this group were interviewed in a focus group discussion to capture collective norms and areas of agreement or disagreement.

† All stakeholders for these groups were interviewed only in key informant interviews for detailed, expert opinions.

## Data Availability

De-identified individual participant data and the study protocol will be made available after publication, for researchers whose proposed use of the data is approved by an independent review committee. Data to be provided for analysis requires a methodologically sound proposal that aligns with the aims of this study. Proposals should be directed to Pratap Kumar at pkumar@strathmore.edu. Data will be shared through a secure institutional server.

## Panel 1: Overarching themes of critical challenges and barriers across the blood transfusion continuum

### Massive deficit in blood availability that does not meet the demand for blood transfusion

#### Negative impact: strain on the health system and health-care workers

“We have a blood shortage. Today there is blood and tomorrow we don’t have any. Even if you find an emergency or urgent case and you need blood.” Focus group, clinicians, 131110

“The main challenge is adequate availability of blood. I wish we could reach that point where we are saying [that] the blood and the blood products are easily available.” Key informant, clinician, 13010384

“[Blood] Is not available most of the time…You see a situation where the relatives must donate blood…Even if there is a [blood drive] camp…you find the demand is too high.” Key informant, clinician, 13010426

### A mismatch between where blood is and where it is needed, reflecting supply chain challenges

#### Negative impact: strain on the health system and health-care workers

“Yesterday, we didn’t have blood…there were no vehicles for the blood, so we were forced to go to our administrator to ask for a vehicle from somewhere else to go and search for blood.” Key informant, administrator, 13010301

“After doing a CS [caesarean section] delivery, a mother experienced postpartum haemorrhage. We needed blood but [it was] not available…It forced us to refer [transfer] the mother because we could not get blood on time. The mother succumbed in the middle of the road, I think a hundred kilometres from my facility…she died of postpartum haemorrhage.” Key informant, clinician, 13010947

“If the patient is bleeding, we come [together] as a team, we ask ourselves ‘Where we can get this blood in other facilities and places?’” Focus group, clinicians, 131110

### An unrealistic reliance on family replacement donors

#### Negative impact: reliance on family replacement donors

“Because we have had situations where relatives go and donate, the blood comes to the inpatient lab but maternity has an emergency…You can’t say no. Paediatrics have an emergency. So my patient knows three relatives donated but [yet] they can’t be transfused.” Focus group, clinicians, 131086

“We were really looking to transfuse a patient a couple of pints, but we’re only able to get one pint of blood. And that was whole blood, we preferred to give platelet transfusion…we had to ask the relatives to come and be matched. Unfortunately, they did not match the patient’s blood group. We had a difficult time because they’re coming from quite a distance. We had a difficult time finding people who could match the patient.” Focus group, clinicians, 131011

“If the patient requires to go to the theatre [surgery], we can’t get the blood. We normally ask the relatives to donate.” Key informant, clinician, 13010426

#### Negative impact: pressures on families for blood donation

“My mom was supposed to undergo major surgery…the hospital says they cannot release blood before it is replaced. The situation where everybody is far, you are the only one present, you are told that [blood] can’t be given…and surgery is a must. You are told it must be done the quickest time possible…I was very mad at that hospital and even now I hate it. When I met that doctor I…tell him ‘You wanted my mother to die, she will not die, I donated blood so she is not dying.’” Focus group, community, 131169

“There was a time [when the] shortage was so bad that you had to know somebody to get blood. I think I have had relatives calling me to talk with somebody in the blood bank so that they can have blood available for a relative’s surgery.” Key informant, clinician, 13010053

### Blood shortages impact patient care and lead to poor health outcomes

#### Negative impact: strain on the health system and health-care workers

“I’m [a] doctor…I’m calling from this ward [needing] units of blood. If I don’t [call] then we will just have a situation where…the patient can wait up to a week to be transfused.” Focus group, clinicians, 131086

“If there are no relatives to donate, we end up cancelling the surgery. The patient stays in the ward and waits for the blood.” Key informant, clinician, 13010426

“It is more initiative than anything for you to get [enough blood] because it comes from a lot of frustration of seeing your patients suffer a lot.” Focus group, clinicians, 131086

### Concern for patient outcomes causes stress to the health-care system

#### Negative impact: strain on the health system and health-care workers

“We’ll lose many babies…I can tell you there are days I wake up and I go to my ward and we have lost seven babies in one morning…they will keep quiet and everybody lives like nothing has happened…if the blood was there, maybe they would have had a chance.” Key informant, clinician, 13010053

“Sometimes the urgent cases in the ward, this one came last week she is still in the ward, this one came this week but now she is worsening then she gets blood earlier than the other one who came earlier on. And it’s an emotive issue and relatives who donate are like, ‘Daktari, where are you? Why not my patient?’ And I’m like, ‘I’m not the one who decides that. It’s just the situation.’ Ultimately, you want the best outcome for your patients. But we don’t get to play God.” Focus group, clinicians, 131060

“There are incidents where there was an accident, people [patients] came in and [I] requested for blood and there was no blood. However, [I] had to go to theatre and do the surgery, because if you didn’t stop the bleeding, eventually the patient will succumb. It’s like you are caught between a stone and a hard place, you have to make those decisions.” Key informant, clinician, 13010574

### Knowledge, attitudes, perceptions, and beliefs about transfusion and donation differ by site and influence the willingness of donors to donate blood and providers to prioritise patients for transfusion

#### Negative impact: low public willingness to donate blood

“I hear from friends and relatives every day about someone needing a transfusion…Helping someone is what motivates me.” Focus group, community, 131144

“Now maybe if you were sensitising people in the villages, you know they are the ones who donate blood…you do campaigns so that people can stop having that mentality that blood is sold. And when people volunteer to donate blood, [the] blood will be a lot.” Key informant, community, 13010715

“Sometimes we do an assessment of cases, which [ones] can wait. We give [blood transfusion] if it’s an obstetric emergency…obviously, that’s the case that you wouldn’t wish to wait as compared to maybe a cancer patient in the ward.” Key informant, clinician, 13010384

### Lack of equipment and resources limits the ability to collect, process, store, transport, and transfuse blood

#### Negative impact: lack of resources

“If the machines in [testing facility] break down, then, samples have to go to Nairobi for screening, so there [are] artificial shortages, you have the blood, your fridge is full of blood, but you cannot transfuse till you have the [test] results.” Key informant, clinician, 13010988

“I’m ashamed to say that’s still a challenge, because we ran out of the blood giving sets [blood administration sets]. Yeah, there is a time we had blood and in nursery we could not transfuse for a whole month because they just can’t get the right [blood administration] set for the neonates to use.” Key informant, clinician, 13010053

“We are also understaffed…We only have three lab techs who are handling the whole hospital. We have very few nurses and you know this is something that has to be observed when you are giving [blood].” Key informant, clinician, 13010400

## Panel 2: Facilitator and proposed innovation themes across the blood transfusion continuum

### Facilitator: health-care providers personally advocate for blood

#### Negative impact: strain on the health system and health-care workers

“Once that blood is donated, you actually have to follow up with the person in charge from the blood bank because there’s usually a miscommunication…so it is my own effort…I have a direct contact with the person in charge of the blood bank.” Focus group, clinicians, 131110

“You will have to go there [to the lab] personally for them to see the situation is dire. Once you leave that place, there is another person who comes following a different issue and it’s urgent. So, you’ll find that there are at times you have to stay there…you watch and take the blood yourself.” Focus group, clinicians, 131086

“I [clinician] had a very sick patient. We shared the same blood group. So, I had to donate [blood].” Focus group, clinicians, 131128

### Facilitator: develop networks and informal supply chains through collaboration

#### Positive impact: collaboration and cooperative actions

“Basically, [we] outsource [blood] from other facilities…and then escalate. We use our facilities that are around [here] then also from other places.” Key informant, clinician, 13010160

“We need these blood groups, we need this blood product, so if the lab doesn’t have that blood product, [we] are to scout around the county and then escalate.” Key informant, clinician, 13010160

“Having someone that we can call, you know, that’s a positive. Because [otherwise] we’d actually get into a situation where we just look at each other in the lab and there is no way forward…” Focus group, clinicians, 131086

“Coordination is the most important thing and of course teamwork at the satellite and [with] other stakeholders.” Key informant, administration, 13010616

### Facilitator: relationships facilitate blood donation

#### Positive impact: community mobilisation efforts

“To me it was a good experience because it was my child who I was supposed to donate blood for him, the only boy, and he was sickly.” Focus group, community, 131169

“I was glad when I received blood, which was donated by somebody and now it was my turn to replace it, so I didn’t see anything bad with it.” Focus group, community, 131169

“When someone says they need blood, I will donate…for me I can donate to anyone, a friend of mine, we will get along.” Key informant, community, 13010855

### Facilitator: resources

#### Positive impact: resources to improve logistics

“The national blood transfusion services have been of great help to us. They are the ones who organised [blood drive] and they really supported the entire operation right from fuelling the vehicle, paying the allowances to the officers, giving the blood bags and everything.” Key informant, administration, 13010616

“There is a budget the county has given…so the county is responsible for ensuring that the blood transfusion is functioning.” Key informant, administration, 13010616

### Facilitator: schools support blood donation

#### Positive impact: blood donation in schools

“During school holidays when students are not in school…blood donation is affected.” Key informant, clinician, 13010426

“We still have very few people donating blood. Most of them are students from colleges and students from high schools.” Key informant, clinician, 13010160

### Innovation: increase public education to improve awareness of voluntary blood donation

#### *Positive impact:* community mobilisation efforts

“For me if they just tell me I’ll be okay health wise, just to encourage me that blood that you have donated will not have any [ill] effect because we have already assessed you, so you are good to donate this blood…So, for me once I’m assured of my health safe-wise, I’ll do it.” Focus group, community, 131169

“In my opinion it’s just education…When they are educated, they will understand.” Focus group, community, 13010814

“All in all, what I can say is that what we need is just empowerment. Sometimes you came to teach me the importance of donating blood. There are some people who are willing to donate blood, but they haven’t been empowered well.” Focus group, community, 131169

“We can create public awareness through mass media, radio, TV, so that many people, even the old will get that information. We can also have pamphlets. These small pamphlets we can give them out in churches, administrators, the chiefs and so on, at least those ones in those committees can reach many people.” Focus group, community, 131151

“It is much easier to mobilise these people; it is a strength that they haven’t realised. They can come here to the rural area and try to mobilise; they will get more people than always going to the traditional donors, the students.” Key informant, administration, 13010400

### Innovation: management of resources

#### Positive impact: resources to improve logistics

“I think those will be things which will be best managed at a more central point, would take the burden off the facility. Then [there are] also issues of resources in stocking, equipment, vehicles, standards and regulations, operating procedures, the sorts of things which [centrally] standardised and set.” Key informant, administration, 13010988

“We need to go out and collect the same blood, which requires a lot of resources. And the need [to] process the same blood this requires expertise [of] technical staff.” Key informant, clinician, 13010202

“We can get the blood but the shortage of reagents for screening [blood testing] has been a paradox.” Key informant, clinician, 13010053

### Innovation: establish hospital-based blood bank with donation, screening, and cross-matching capabilities

#### Positive impact: collaboration and cooperative actions

“If we [local facility] were testing [blood] it would be maybe faster and maybe it would lessen the waiting period before the patient receives the blood.” Key informant, administration, 13010467

“I wish that counties would have capacities to establish their own screening centres, that would really be like what Nakuru has done, that will really solve a lot of problems.” Key informant, administration, 13010335

“So, if we have a regional centre here, it will be much, much better in terms of efficiency in terms of time, and then even coordination. It will be important if they would be given a processing unit of their own then it would really save more lives.” Key informant, clinician, 13010111

### Innovation: improve sharing of blood resources and supplies between hospitals

#### Positive impact: collaboration and cooperative actions

“We work as a team, we really need to come together, put our minds together and reason for this patient because at the end of the day, the patient is the one who’s benefiting. The MO [Medical Officer] has been mentioned, the central role of the nurse has been mentioned, and the other team members that play a part in this difficult decision making…We reason together…We work as a team; it is very important.” Focus group, clinicians, 131110

“You can either borrow blood or refer the patient. [You] weigh the two options. You can borrow [blood] instead of referring the patient.” Focus group, clinicians, 131110

### Innovation: hospital transfusion committees

#### Positive impact: collaboration and cooperative actions

“He [lab manager] has put [together] a committee [and] assigned his colleagues–you are in charge of this, you are in charge of this, you are in charge of this—anything that happens there; even if the standards are low…if something happens there, that is supposed to be told, they inform [everyone]. Every Monday, they have a meeting where they review their services, through the blood committee, the blood personnel, commodity manager within the laboratory, he gives the report and the lab manager, what I usually see him doing is he brings the report to the administration block, whereby they share if something needs to be done.” Key informant, administration, 13010301

“The other thing is strengthening our HTC [Hospital Transfusion Committee]…They are very crucial because they may be the ones that oversee all the transfusion at the facility level. These committees are very important for that task.” Key informant, administration, 13010616

### Innovation: establish standard processes and quality improvement

#### *Positive impact:* standardisation and improvement

“I remember when I worked at [facility], I used to go to the lab, you will find they have racks that are labelled; Monday, Tuesday, Wednesday, Thursday something like that. Let’s say I bring my sample on Monday; it has [a] Monday space. That way, it is easy to follow up.” Key informant, clinician, 13010426

“We’re supposed to have Continuous Quality Assurance activities running for routine work at the lab and the clinical area. My job is to make sure standards are maintained and continuously improved. And are…in line with any laws regulations that change around this office…and are continuous.” Key informant, administration, 13010988

“There [are] things called SOPs, standard operating procedures. SOPs are standard[ised] all over. There are systems in place. Everything is okay. SOPs have been standard all over. There are conferences staff attend for best practice. There are managers and supervisors in the public sector going round checking on quality.” Key informant, clinician, 13010483

## Research in context

### Evidence before this study

We searched PubMed and Google Scholar using the terms “blood transfusion”, “blood donation”, or “blood availability” and “sub-Saharan Africa”, for original research articles published in English from January, 2000, to December, 2020. Of the approximately 800 articles returned, only 12 used qualitative methods to understand diverse perspectives of essential participants in blood transfusion, with most focusing on blood donors. There was a paucity of literature describing socioecological factors across the blood transfusion continuum from vein to vein, systemic challenges to blood availability (besides donor mobilisation), or measures taken to improve gaps from the Kenyan perspective.

### Added value of this study

This is the largest qualitative study of its kind in sub-Saharan Africa synthesising diverse multidisciplinary perspectives on blood availability. Findings from this study, based in Kenya, might be relevant to many low-income and middle-income countries seeking to address gaps in blood availability. The overall function of the health systems is adversely impacted by lack of blood availability, contributing to poor patient outcomes, clinician self-efficacy, and capacity utilisation. Innovations proposed to address blood availability range from individual actions, community engagement, health systems enablement, and policy change.

### Implications of all the available evidence

This study paints a rich picture of the complexity of the blood system continuum and contextualised opportunities to improve the availability of blood transfusion. Importantly, no singular cause is implicated in the lack of blood availability nor is any sole authority situated to comprehensively mediate the complex system dynamics across the continuum. Multilevel changes are necessary to overcome challenges in blood availability, including individual behaviours, community participation, logistics orchestration and forecasting, and health systems integration. Participatory approaches engaging community stewardship and shared governance might contribute to greater blood transfusion availability.

## References

[1] Delaney M, Telke S, Zou S, The BLOODSAFE program: building the future of access to safe blood in sub-Saharan Africa. Transfusion 2022; 62: 2282. 10.1111/trf.17091 PMC9643608 36173295

[2] Roberts N, James S, Delaney M, Fitzmaurice C. The global need and availability of blood products: a modelling study. Lancet Haematol 2019; 6: e606–15. 10.1016/S2352-3026(19)30200-5 31631023

[3] Barro L, Drew VJ, Poda GG, Blood transfusion in sub-Saharan Africa: understanding the missing gap and responding to present and future challenges. Vox Sang 2018; 113: 726–36. 10.1111/vox.12705 30221365

[4] Geller SE, Koch AR, Garland CE, MacDonald EJ, Storey F, Lawton B. A global view of severe maternal morbidity: moving beyond maternal mortality. Reprod Health 2018; 15 (suppl 1): 98. 10.1186/s12978-018-0527-2 PMC6019990 29945657

[5] Say L, Chou D, Gemmill A, Global causes of maternal death: a WHO systematic analysis. Lancet Glob Health 2014; 2: e323–33. 10.1016/S2214-109X(14)70227-X 25103301

[6] Raykar NP, Raguveer V, Abdella YE, Innovative blood transfusion strategies to address global blood deserts: a consensus statement from the Blood Delivery via Emerging Strategies for Emergency Remote Transfusion (Blood DESERT) Coalition. Lancet Glob Health 2024; 12: e522–29. 10.1016/S2214-109X(23)00564-8 PMC10882207 38365422

[7] Oduor M Progress in blood transfusion services in Kenya from the 1980’s: a review. East Afr Med J 2009; 86 (suppl 12): S98–101. 21591518

[8] Kipkulei JC. Documentation of the clinical blood transfusion practice at a teaching and referral hospital in western Kenya. Am J Health Res 2019; 7: 8–11.

[9] Raykar NP, Makin J, Khajanchi M, Assessing the global burden of hemorrhage: the global blood supply, deficits, and potential solutions. SAGE Open Med 2021; 9: 20503121211054995. 10.1177/20503121211054995 PMC8591638 34790356

[10] Munoz-Valencia A, Aridi JO, Barnes LS, Protocol: identifying policy, system, and environment change interventions to enhance availability of blood for transfusion in Kenya, a mixed-methods study. BMC Health Serv Res 2023; 23: 963. 10.1186/s12913-023-09936-0 PMC10486046 37679772

[11] Damschroder LJ, Aron DC, Keith RE, Kirsh SR, Alexander JA, Lowery JC. Fostering implementation of health services research findings into practice: a consolidated framework for advancing implementation science. Implement Sci 2009; 4: 50. 10.1186/1748-5908-4-50 PMC2736161 19664226

[12] Dei-Adomakoh Y, Asamoah-Akuoko L, Appiah B, Yawson A, Olayemi E. Safe blood supply in sub-Saharan Africa: challenges and opportunities. Lancet Haematol 2021; 8: e770–76. 10.1016/S2352-3026(21)00209-X 34481544

[13] Smith A, Matthews R, Fiddler J. Blood donation and community: exploring the influence of social capital. Int J Soc Inq 2011; 4: 45–63.

[14] Mohammed AD, Ntambwe P, Crawford AM. Barriers to effective transfusion practices in limited-resource settings: from infrastructure to cultural beliefs. World J Surg 2020; 44: 2094–99. 10.1007/s00268-020-05461-x PMC7266790 32157404

[15] Adomakoh YD, Olayemi E, Asamoah-Akuoko L, Impact of iron supplementation in anemic voluntary first-time blood donors-results of a pilot trial in Ghana. Blood 2023; 142 (suppl 1): 1290. 10.1111/trf.18082 PMC11750598 39614766

[16] Jacobs JW, Bates I, M’baya B, Ensuring a safe and sufficient global blood supply. N Engl J Med 2024; 391: 1079–81. 10.1056/NEJMp2403596 39115103

[17] Jacobs JW, Bates I, Cohn CS, The global blood donation index: an imperfect measure of transfusion need. Lancet 2024; 404: 988–90. 10.1016/S0140-6736(24)01550-2 PMC12716954 39244275

[18] Ozawa S, Ozawa-Morriello J, Perelman S, Thorpe E, Rock R, Pearse BL. Improving patient blood management programs: an implementation science approach. Anesth Analg 2023; 136: 397–407. 10.1213/ANE.0000000000006273 36638516

[19] Santos WJ, Graham ID, Lalonde M, Demery Varin M, Squires JE. The effectiveness of champions in implementing innovations in health care: a systematic review. Implement Sci Commun 2022; 3: 80. 10.1186/s43058-022-00315-0 PMC9308185 35869516

[20] Bates I, Owusu-Ofori S. Blood transfusion. In: Cook GC, Zumla AI, eds. Manson’s tropical diseases, 23rd edn. Elsevier, 2014: 229–34.

[21] Metcalf GS. In: Social systems and design. vol 1. Tokyo: Springer Japan; 2014.

[22] Li N, Pham T, Cheng C, Blood demand forecasting and supply management: an analytical assessment of key studies utilizing novel computational techniques. Transfus Med Rev 2023; 37: 150768. 10.1016/j.tmrv.2023.150768 37980192

[23] Thakur SK, Sinha AK, Negi DK, Singh S. Forecasting demand for blood products: towards inventory management of a perishable product. Bioinformation 2024; 20: 20–28. 10.6026/973206300200020 PMC10859947 38352907

[24] Loua A, Sonoo J, Musango L, Nikiema JB, Lapnet-Moustapha T. Blood safety status in WHO African region countries: lessons learnt from Mauritius. J Blood Transfus 2017; 2017: 1970479. 10.1155/2017/1970479 PMC5664371 29181226

[25] Jayaram A, Dutta R, Kim EK, Alternative strategies for emergency blood transfusion in low-resource settings: a scoping review. Transfusion 2024; 64: 1350–61. 10.1111/trf.17838 38742837

[26] Saner M, Wilson J. Stewardship, good governance and ethics. Institute on Governance Policy Brief No 19. 2003. https://ssrn.com/abstract=1555815 (accessed April 26, 2025).

[27] Van Puyvelde S, Caers R, Du Bois C, Jegers M. The governance of nonprofit organizations. Nonprofit Volunt Sector Q 2012; 41: 431–51.

[28] Carlson V, Chilton MJ, Corso LC, Beitsch LM. Defining the functions of public health governance. Am J Public Health 2015; 105 (suppl 2): S159–66. 10.2105/AJPH.2014.302198 PMC4355716 25689187

[29] Gonçalez TT, Di Lorenzo Oliveira C, Carneiro-Proietti ABF, , and the NHLBI Retrovirus Epidemiology Donor Study-II (REDS-II), International Component. Motivation and social capital among prospective blood donors in three large blood centers in Brazil. Transfusion 2013; 53: 1291–301. 10.1111/j.1537-2995.2012.03887.x PMC3542409 22998740

