# Supplementary material for: Exploration of the vein to vein blood transfusion system in three Kenyan counties: a qualitative study
Source: Lancet Glob Health. Author manuscript; Available in PMC 2026 Oct 10. (PMC13653559; doi:10.1016/j.langlo.2026.103968)

THE LANCET
Global Health

## Supplementary appendix 1

This appendix formed part of the original submission and has been peer reviewed.
We post it as supplied by the authors.

Supplement to: Barnes LS, Makin J, Beneitez JP, et al. Exploration of the vein to vein blood transfusion system in three Kenyan counties: a qualitative study. *Lancet Glob Health* 2026.
https://doi.org/10.1016/j.langlo.2026.103968

Web Appendix Table 1: Purposive sampling of participants across the blood transfusion continuum

| Participants (N) % | Role | Title/Position | Nakuru (N) | Turkana (N) | Siaya (N) |
|---|---|---|---|---|---|
| Administration (24) 20% | Government Official | County Minister/ Director | 1 | 1 | 1 |
| | Level I-II Hospital* | Superintendent/Chief Officer/Director | 2 | 2 | 2 |
| | Level III-IV Hospital* | Superintendent/Chief Officer/Director | 2 | 2 | 2 |
| | National blood service | Director/Manager | 3 | | |
| | Regional blood service | Director/Manager | 1 | 1 | 1 |
| | Satellite blood service | Director/Manager | 1 | 1 | 1 |
| | | | | | |
| Clinician (69) 57.5% | Consultant | Oncologist/Nephrologist | 2 | 2 | 2 |
| | Consultant | Pediatrician/Internist | 2 | 2 | 2 |
| | Consultant | OBGYN/Surgeon | 2 | 2 | 2 |
| | Medical Officer | General | 6 | 6 | 6 |
| | Clinical Officer | Outpatient Provider | 2 | 2 | 2 |
| | Inpatient Nurse | Oncology/Dialysis Ward | 2 | 2 | 2 |
| | Inpatient Nurse | Pediatric/Medical Ward | 2 | 2 | 2 |
| | Inpatient Nurse | Obstetric/Surgical Ward | 2 | 2 | 2 |
| | Outpatient Nurse | Oncology/General Outpatient Clinic | 3 | 3 | 3 |
| Community (27) 22.5% | Donor | Family Replacement Donor / Volunteer | 3 | 3 | 3 |
| | Patient | Oncology Ward | 2 | 2 | 2 |
| | Patient | Pediatric/Medical Ward | 2 | 2 | 2 |
| | Patient | Obstetric/Surgical Ward | 2 | 2 | 2 |
| Planned Participants >= 120 | | | | | |

*Kenya categorizes hospitals as levels I-IV; level I=health center, level II =sub-county hospital, level III=county referral hospital, level IV=national referral hospital.

Web Appendix Table 2: Interview and discussion topic domains with example questions.

| Research Question | Construct | Subconstruct | Sample Interview Questions | CFIR Domain / Construct | Data Source | Measures | Integration |
|---|---|---|---|---|---|---|---|
| Identify challenges across the blood continuum | Respondent Context | Role, facility level, patient population | What is your role, years of service, education, and facility level? Which county and type of facility do you work in? What patient populations do you serve? | Characteristics of Individuals – Knowledge & Role | KII | Role variation across facilities | Contextual analysis |
| Identify system perceptions | Overall System Perception | System performance trends | What is your overall impression of blood availability and transfusion services? Have you noticed improvements or declines over time? Are there regional differences? | Inner Setting – Implementation Climate | KII, FGD | Patterns across counties | Regional comparisons |
| Identify donor supply challenges | Blood Donation & Recruitment | Donor sources, recruitment strategies | What are the main sources of blood? How are donors recruited and retained? What are barriers to donation? How can donation rates be improved? | Outer Setting – Patient Needs & Resources | KII, FGD | Frequency of barriers vs facilitators | Inform donor recruitment interventions |
| Identify donor facilitators | Facilitators to Donation | Motivation, self-efficacy, community engagement | What factors encourage people to donate blood? What strategies have worked well to recruit donors? How effective are donor campaigns or outreach programs? | Characteristics of Individuals – Self-efficacy / Motivation | FGD, KII | Facilitator themes | Intervention design |
| Identify operational challenges | Collection & Processing | Testing workflows, logistics | How is blood collected and processed? What tests are conducted? What challenges exist in testing and quality assurance? What happens if problems are identified? | Inner Setting – Structural Characteristics | KII* | Process bottlenecks | Workflow modeling |
| Identify supply chain challenges | Storage & Distribution | Inventory management, transport | Where is blood stored and under what conditions? How is blood transported? Are tracking or inventory systems used? What are delivery barriers? | Inner Setting – Available Resources | KII | Storage capacity variation | Supply chain modeling |
| Identify clinical demand drivers | Clinical Utilization | Indications, transfusion thresholds | For which conditions is blood most frequently requested? What transfusion thresholds are used? Which patient groups consume the most blood? | Outer Setting – Patient Needs | KII | Patterns of utilization | Demand modeling |
| Identify prioritization mechanisms | Ordering & Allocation | Decision rules | How are blood orders placed? Who prioritizes allocation? Are certain patients prioritized? | Inner Setting – Workflow & Decision Processes | KII | Allocation practices | Resource allocation modeling |
| Identify supply gaps | Delays & Stockouts | Turnaround time, shortages | What happens during shortages? How long does it take to receive blood? How do delays impact patients? | Inner Setting – Available Resources | KII, FGD | Delay duration and narratives | Patient outcome analysis |
| Estimate demand vs supply | Supply-Demand Gap | Units requested vs supplied | How many units are requested per day/week/month? How many patients fail to receive needed blood? Would demand increase with unlimited supply? | Outer Setting – Patient Needs | KII | Quantitative estimates | Model inputs |
| Capture patient and donor perspectives | Patient & Donor Experience | Access, perceptions, cultural beliefs | Describe the experience of donating or receiving blood. What barriers exist? What improvements are needed? | Characteristics of Individuals – Beliefs | FGD, KII | Experience themes | Behavioral intervention design |
| Identify system barriers | Barriers Across Continuum | Policy, logistics, resource constraints | What are the major barriers in donation, processing, delivery, and administration? What operational or policy challenges exist? | Inner / Outer Setting | KII, FGD | Barrier frequency | Prioritize interventions |
| Identify system facilitators | Facilitators Across Continuum | Infrastructure, partnerships, leadership | What parts of the blood system work well? What resources or systems help ensure blood availability? | Inner Setting – Available Resources | KII | Facilitator themes | Strength-based system analysis |

| | | | | | | | |
|---|---|---|---|---|---|---|---|
| Identify system resilience | Workarounds | Informal strategies | What informal strategies are used to secure blood? How do facilities manage shortages? | Process – Adaptation | KII | Types of workarounds | Innovation identification |
| Examine governance structures | Governance & Policy | Oversight, regulation | Which agencies oversee blood services? How are policies or technologies approved? How are errors reported and managed? | Outer Setting – External Policy & Incentives | KII | Governance mapping | Policy recommendations |
| Assess quality and safety | Quality & Safety | Monitoring, guidelines | How is transfusion safety ensured? Are standard transfusion guidelines followed? | Inner Setting – Quality Monitoring | KII | Safety practices | Implementation improvement |
| Identify innovations | System Improvements | Adaptations, innovations | What improvements are needed in the blood system? What innovations would you prioritize? | Process – Reflecting & Evaluating | KII, FGD | Innovation themes | Intervention design |
| Assess implementation feasibility | Technology Innovations | Mobile/social media recruitment | How feasible would mobile or social media technologies be for donor recruitment? | Intervention Characteristics – Relative Advantage / Adaptability | KII | Perceived feasibility | UH3 implementation planning |

*Key Informant Interview KII or Focus Group Discussion FGD

Web Appendix

Figure: Qualitative data analysis followed an iterative approach in three successive rounds of analysis. The coding structures (blue) were refined by preliminary analysis of 15 transcripts (orange), followed by a comprehensive analysis of all transcripts. Emergent codes, patterns, and themes benefited from stakeholder validation.

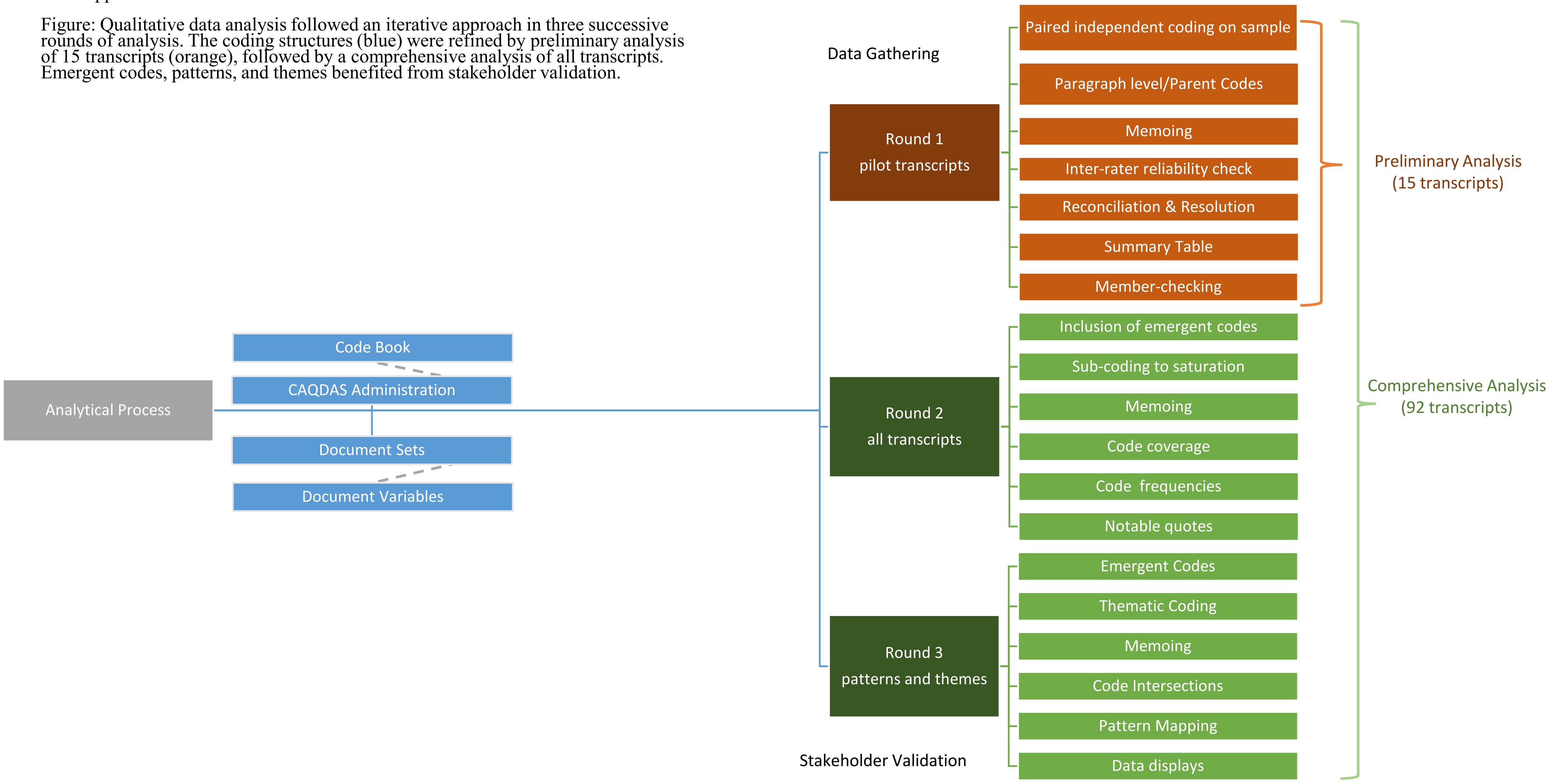

Supplement: Supplementary file 1 [file NIHMS2209795-supplement-supplemental_appendix_1.pdf]
