# Supplementary material for: Exploration of the vein to vein blood transfusion system in three Kenyan counties: a qualitative study
Source: Lancet Glob Health. Author manuscript; Available in PMC 2026 Oct 10. (PMC13653559; doi:10.1016/j.langlo.2026.103968)
Supplement: Supplementary file 2 [file NIHMS2209795-supplement-supplemental_appendix_2.pdf]

THE LANCET
Global Health

Supplementary appendix 2

This appendix formed part of the original submission and has been peer reviewed.
We post it as supplied by the authors.

Supplement to: Barnes LS, Makin J, Beneitez JP, et al. Exploration of the vein to vein blood transfusion system in three Kenyan counties: a qualitative study. *Lancet Glob Health* 2026.
https://doi.org/10.1016/j.langlo.2026.103968

Equitable Partnership Declaration

If any questions do not apply to your study, please indicate "N/A" for "not applicable.
For more information on how to complete this form see the Information for Authors document.

Researcher considerations

1. Please detail the involvement that researchers who are based in the country or countries of study had during a) study design; b) clinical study processes, such as processing blood samples, prescribing medication, or patient recruitment; c) data interpretation; and d) manuscript preparation, commenting on all aspects. If they were not involved in any of these aspects, please explain why.

   This should include a thorough description of their leadership roles in the study. Are local researchers named in the author list or the acknowledgements, or are they not mentioned at all (and, if not, why)? Please also describe the involvement of early career researchers based in the location of the study. Some of this information might be repeated from the Contributors section in the manuscript. Note: we adhere to ICMJE authorship criteria for naming authors on a paper.

**a) Study design:**<br><br>The study design involved multiple collaborators who are residents of Kenya, including the senior author, Pratap Kumar. The following co-authors are residents of Kenya: Jana MacLeod, Cindy M Makanga, Tecla Chelagat, Abdirahaman Musa, Robert Kamu Kaburu, Carolyne Njoki, and Pratap Kumar.
**b) Clinical study processes:**<br><br>Kenyan residents Jana MacLeod, Cindy M Makanga, Tecla Chelagat, Abdirahaman Musa, Robert Kamu Kaburu, Carolyne Njoki, Cavin Otieno Opiyo, and Pratap Kumar performed recruitment of participants, consenting, and data collection. Further, they were involved in member checking and stakeholder validation.<br><br>Strathmore University (a Kenyan university) housed the research infrastructure in collaboration with other international academic institutions and public health organizations. Informed consent forms and their local language translations were approved by a nationally accredited IRB in Kenya. The research was led by Kenyan researchers based in a Kenyan university with staff hailing from and based in the three counties where the research was conducted.
**c) Data interpretation:**<br><br>Kenyan residents Jana MacLeod, Cindy M Makanga, Tecla Chelagat, Abdirahaman Musa, Robert Kamu Kaburu, and Pratap Kumar participated in qualitative analysis and interpretation.
**d) Manuscript preparation:**<br><br>Kenyan residents Jana MacLeod, Cindy M Makanga, Tecla Chelagat, Abdirahaman Musa, Robert Kamu Kaburu, Carolyne Njoki, Cavin Otieno Opiyo, and Pratap Kumar participated in manuscript preparation conforming with ICMJE.

2. How was funding used to remunerate and enhance the skills of researchers in the countries of study? And how was funding used to improve research infrastructure at the study sites?

   Potentially effective investments into long-term skills and opportunities within local institutions could include training or mentorship in analytical techniques and manuscript writing, opportunities to lead all or specific aspects of the study, financial remuneration rather than requiring volunteers, and other professional development and educational opportunities.

   Improvements to research infrastructure could include funding extended trial designs (eg, platform trials), establishment of long-term contracts for research staff, building research facilities, and setting up local control of funding allocation.

<table>
<tr><td>Skills:

For this qualitative exploratory manuscript, extensive training and mentorship developed research skills within the team, including but not limited to:

How to conduct literature reviews
How to integrate theoretical frameworks
How to create a sampling plan
How to develop and refine the codebook used for the qualitative analysis
Training in interview techniques
How to run focus group discussions
Training in Human Subjects Research Practices and Good Clinical Practices
Training in computer-assisted qualitative analysis
How to run workshops to iteratively identify and refine emerging themes
How to perform member-checking with participants
How to engage stakeholders to validate findings
How to develop and submit an abstract to a scientific meeting
How to write a poster
How to give an oral presentation
How to prepare a manuscript
How to generate responses to reviewer comments

These efforts helped junior researchers to refine and apply skills by developing scientific posters, oral presentations, and ultimately, manuscripts. Five posters were accepted for the Africa Society for Blood Transfusion (AfSBT) International Congress in Namibia (2026). Additionally, Ms Cindy M Makanga received the Emerging Transfusion Researcher Award for her oral presentation at the AfSBT International Congress in 2026 as an outcome of these efforts.</td></tr>
<tr><td>Research infrastructure:

The qualitative research team developed important infrastructure, including but not limited to:

Data collection tools
Data management agreements
Data management plans
Memoranda of Understanding with key partner organizations</td></tr>
</table>

Collaborations with ministries of health, including the Kenya National Transplant and Blood Service
Computer-assisted qualitative data analysis licenses (MAXQDA 2022)

3. How did you safeguard the researchers who implemented the study?

Please describe how you guaranteed safe working conditions for study staff, including provision of appropriate personal protective equipment, protection from violence, and prevention of overworking.

Care was taken to ensure safety of the study team including biohazardous safety practices for working in clinical and laboratory settings, personal protective equipment, good clinical practices, and good participatory research practices. Weekly study team meetings were held to discuss challenges and develop strategies to mitigate difficulties and recognize best practices.

Benefits to the communities and regions of study

4. How does the study address the research and policy priorities of its location?

How were the local priorities determined and then used to inform the research question? Who decided which priorities to take forward? Which elements of the study address those priorities?

This study directly reflects the views of local participants highlighting challenges, facilitators, and innovations within the blood transfusion continuum. The research team worked with healthcare administrators (hospital, laboratory and blood bank) in each of the counties as part of developing the aims and protocol of this research. A widely represented, participative, stakeholder workshop was held as part of this research that included patients, donors, healthcare providers (diverse cadres), administrators and policymakers. The hybrid inductive and deductive analysis enabled the identification of emergent themes less prominent in the current literature yet highly relevant to the local context. Thus, recommendations expressed by the participants are likely more actionable as policy and research priorities.

5. How will research products be shared in the community of study?

For instance, will you be providing written or oral layperson summaries for non-academic information sharing? Will study data be made available to institutions in the region(s) of study? The Lancet Group *encourages authors to translate the summary (abstract) into relevant languages after paper editing; do you intend to translate your summary?*

Preliminary findings have already been shared with stakeholders in all three counties where this study was conducted. Findings from this study are part of follow-on research in the three counties (https://clinicaltrials.gov/study/NCT06142825). Dissemination of these findings will

be shared with national and local policymakers, governing bodies, healthcare facilities, and public health organizations through stakeholder learning and sharing sessions. We will translate the abstract into local languages of Turkana, Swahili, and Luo, for easier accessibility within the region studied. Further, this research expanded into a novel intervention implemented as a cluster randomized controlled study, CoBAnK, reporting results later in 2026. The Kenya National Blood Transfusion and Transplant Service was a research participant and collaborated throughout the study to develop a deeper understanding of how these findings could inform blood availability improvements within the country.

How were individuals, communities, and environments protected from harm?

a) *How did you ensure that sensitive patient data were handled safely and respectfully? Was there any potential for stigma or discrimination against participants arising from any of the procedures or outcomes of the study?*

The study team maintained a data management plan and procedures to protect research data in a secure and confidential manner. Once consented, participants were de-identified using a unique participant number rather than by name or other individually identifiable characteristic. Interviews and focus group discussion were conducted in comfortable, private settings conducive to sharing opinions.

b) *Might any of the tests be experienced as invasive or culturally insensitive?*

No clinical tests were performed as part of this qualitative study.

c) *How did you determine that work was sensitive to traditions, restrictions, and considerations of all cultural and religious groups in the study population?*

The persons participating in the study were selected for their experiences with blood donation and transfusion in the Kenyan context. Local language translations were conducted by persons living and working in the three counties. They were approved by local IRBs and pilot tested for understanding. The interview and focus group guides were designed to collect information about these experiences in a neutral, non-judgmental manner. The study personnel conducting the interviews were Kenyan residents trained in qualitative techniques allowing for cultural sensitivity and awareness within the study population.

d) *Were biowaste and radioactive waste disposed of in accordance with local laws?*

N/A—this qualitative study did not directly generate biowaste or radioactive waste.

e) *Were any structures built that would have impacted members of the community or the environment (such as handwashing facilities in a public space)? If so, how did you ensure that you had appropriate community buy-in?*

No

f) *How might the study have impacted existing health-care resources (such as staff workloads, use of equipment that is typically employed elsewhere, or reallocation of public funds)?*

This qualitative study accessed participants within healthcare facilities and surrounding communities (e.g., blood donors, patients, officials). Consenting described the study, including time commitments and associated risks/benefits. As described in the consent approved by the local ethics committee, participants were reimbursed for their time and expenses. There was no reallocation of public funds or equipment associated with this qualitative study.

6. Confirm that local ethics review was sought, and please provide the approval number. If not sought, please explain why.

The study protocol was reviewed and approved by:

1. The Strathmore University Institutional Ethic Review Committee (reference number #SU-IERC0992/21, #SU-IERC1345/22), which is a local IRB
2. The Kenyan National Commission for Science, Technology & Innovation (license number NACOSTI/P/21/9976, NACOSTI/P/22/17897)
3. The University of Pittsburgh Institutional Review Board (reference number #STUDY21020040).

The protocol was also approved by county health administrations in each of the three counties where the study was conducted

Secondary analyses

7. Have the data analysed in your study been extracted from another source, such as a national survey, rather than being directly collected by the authors of this paper?

No

If the authors of this paper were not involved in data collection, how were the findings interpreted with sufficient contextual knowledge?
The Lancet Group *believe contextual understanding is crucial for informed data analysis and interpretation.*

N/A—the authors were involved in primary data collection.

---

8. Please provide the title (eg, Dr/Prof, Mr/Mrs/Ms/Mx), name, and email address of an author who can be contacted about this statement.

Name: Dr. Linda S Barnes
Email: lbarne23@gmail.com

9. Finally, please provide the title and name of an author from one country of study who has seen and approved this form.

**Name:** Dr. Pratap Kumar (Kenya)
